# Cost-effectiveness analysis of ultrasound-guided Seldinger peripherally inserted central catheters (PICC)

**DOI:** 10.1186/s40064-016-3698-8

**Published:** 2016-12-01

**Authors:** Jianghong Tan, Liping Liu, Jing Xie, Lingli Hu, Qiaolan Yang, Honghong Wang

**Affiliations:** 1 Nursing College of Central South University, Zhuzhou Central Hospital, Room 210, Administration Building, Zhuzhou, 410007 Hunan China; 2 Zhuzhou Central Hospital, Zhuzhou, 410007 Hunan China; 3 Nursing College of Central South University, Yuelu District Tongzipo Road, Changsha, 410013 Hunan China

**Keywords:** Peripherally inserted central catheter, Ultrasound, Seldinger technology, Cost-effectiveness, Cost-effectiveness

## Abstract

**Background:**

Ultrasound-guided cannulation of deep mid-arm veins by a modified Seldinger (US-Seldinger) technique has been demonstrated to yield better puncture success rates and lower postoperative complication rates than direct cannulation of superficial veins near the elbow with a short peripheral cannula and peripherally inserted central catheter (PICC) insertion through the cannula (non-US conventional method). Economic factors have been evaluated across different operators (i.e. nurses, radiologists, and general practitioners) and different venous catheter types (i.e. PICCs vs. central venous catheters). However, to our knowledge, data describing the economic evaluation on the aforementioned modified Seldinger technique are lacking. Hence, the aim of this study was to evaluate the cost-effectiveness of US-Seldinger technique (experimental group) compared with that of the non-US conventional method based on direct vein visualization (control group).

**Results:**

A cohort of 360 subjects were assigned randomly to the experimental and control groups. Cost-effectiveness ratio (CER) analyses indicated that the effectiveness index (EI) for the experimental group was 89.29% (final CER = 3732.75), whereas that for the control group was 59.18% (final CER = 2492.98).

**Conclusion:**

The US-Seldinger technique was found to be more cost-effective than the non-US conventional method. These findings support the use of the former in place of the traditional latter technique as a routine puncture technique and suggest that the update would improve intravenous therapy treatment for patients needing PICCs. This study should serve as a reference for national healthcare policy.

*Trial registration* ChiCTR-TRC-14004993

**Electronic supplementary material:**

The online version of this article (doi:10.1186/s40064-016-3698-8) contains supplementary material, which is available to authorized users.

## Background

Use of peripherally inserted central catheters (PICCs) is increasing worldwide (Parkinson et al. 1998). In recent years, Chinese physicians have been gaining familiarity with the ultrasound-guided Seldinger puncture technique of PICC placement, as an alternative to the cannula needle puncture (CNP) method of casing needle catheterization. However, concerns remain regarding the cost of the ultrasound-guided cannulation of deep mid-arm veins by a modified Seldinger (US-Seldinger) technique, particularly for patients living in poverty without medical insurance for whom the cost may be too burdensome (Robinson et al. 2005; Royer 2001). Furthermore, broader application of this technique has been limited by there being few nurses with sufficient training and unfamiliarity among patients in China (Zhang 2010). Currently, it is used only as a remedial measure specifically for the minority of patients who have poor venous conditions or with whom casing needle catheterization has been unsuccessful (Chen 2003; Chen and Chen 2009; Shen et al. 2008; Shen and Li 2009).

Compared with the direct cannulation of superficial elbow veins with a short peripheral cannula and PICC insertion through the cannula (non-US conventional method), the US-Seldinger technique has been demonstrated to yield better puncture success rates and lower incidence rates of postoperative complications (Schwengel 2004; Malcolm and Robinson 2005; American Diabetes Association 2006). Economic factors have been evaluated across different operators (i.e. nurses, radiologists, and general practitioners) and different venous catheter types (i.e. PICCs vs. central venous catheters) (Schwengel 2004; Malcolm and Robinson 2005). However, to our knowledge, the economics of different PICC puncture methods have not been compared. Therefore, the aim of this study was to compare the cost-effectiveness of the US-Seldinger to that of the non-US conventional method. The results are intended to provide evidence that can be used to support selection of which PICC catheterization method should be adopted by clinical practices and for national health insurance policy making.

## Methods

### Study design, randomization, and participant

This study was a randomized controlled single-blinded trial. From May 2009 to April 2011, patients needing a PICC were recruited in sequence. The inclusion criteria were: (1) 20–60 years of age; (2) expected treatment duration of <6 months; (3) ambulatory; and (4) ability to give consent and express wishes. The exclusion criteria were: (1) prior chemotherapy; (2) PICC replacement over guide wire; (3) PICC was inserted in another site besides the arm (i.e. via external jugular vein or femoral vein, etc.); (4) known or suspected systemic infection, puncture site infection, injury, or history of radiation; (5) superior vena cava syndrome; (6) PICC insertion ipsilateral to breast cancer surgery; and (7) history of radiotherapy near the vena cava. The study was approved by the ethics committee of the local hospital. Informed consent was obtained from each participant before the study.

Pediatric patients were not included for two reasons. Firstly, children require special (smaller) catheters. Secondly, the catheterization process is complicated by the fact that many children have great difficult remaining still and quiet during catheterization, which could have affected several outcome measures of this study, including puncture success rate, bleeding, and pain scores. Conversely, the study did not include patients over 60 years of age due to aging-related declines in blood vessel elasticity (according to ultrasound division statistics in our hospital and our experience), which could also affect the study’s outcome measures.

As shown in Additional file 1: Figure S1, all included patients were divided randomly into two groups: (1) traditional non-US method (control group); and (2) US-Seldinger technique (experimental group). For randomization, the participants were allocated according the last digit of their patient ID numbers; patients with odd and even last digits were placed in the control group and experimental group, respectively. The nurse recorded the catheterization method used, and thus group enrollment, but were not aware of the purpose of the study.

### Sample size estimation

The success rate of the first puncture was taken as the principal outcome measure. The projected group sample size *n* was calculated based on the equations:

$$ n = \left( {\frac{{u_{\alpha } + u_{\beta } }}{\delta }} \right)^{2} \left[ {\pi_{1} (1 - \pi_{1} ) + \pi_{2} (1 - \pi_{2} )} \right] ,\,\;\;{\text{and}}\; \, \delta = \pi_{1} - \pi_{2} $$

where δ is the difference between the expected success rates. We supposed a projected first puncture success rate of 87.5%, based on prior work (Tan and Liu 2010), and supposed a success rate for the US-Seldinger technique of 98.7%, with *α* = 0.05, 1 − *β* = 0.80. Thus, we estimated:

$$ \updelta =  0.987  -  0.875  =  0.112, $$

and calculated *n* as follows:

$$ n = \left( {\frac{1.96 + 1.282}{0.112}} \right)^{2} \left[ {0.987(1 - 0.987) + 0.875(1 - 0.875)} \right] = 102.41 $$

From this calculation, we determined that the sample size should be at least 103 cases. Taking into account the potential for patient drop-off, a total group size of 180 patients was adopted to ensure that the sample size would be adequate to meet the requirements of the study.

### Catheterization methods

Catheter insertion and maintenance was performed by a trained vascular access nurse (J.X.) who was already (American Diabetes Association 2006) familiar with intravenous therapy (American Diabetes Association 2006; Tan and Liu 2010). All patients were fitted with Groshong silicon PICCs (size 4Fr); Bard Access Systems Inc., USA).

For the control group treated with the traditional short peripheral venous catheter puncture method without ultrasound guidance (Additional file 2: Figure S2), elbow blood vessels were first assessed by visualization and palpation guided by clinical experience. An accessible basilica, median cubical, or cephalic vein region was first selected, and then the puncture point was marked with a marker. While the patient’s skin was fixed at the puncture point with the operator’s left hand, a 14G puncture needle was used to pierce the blood vessel at the marked point with the operator’s right hand. Then the stylet was withdrawn and the catheter was placed into the designated location through a stab sheath. The projection position of the PICC tube end was located in the second to third intercostal space; this location was confirmed by bedside chest X-rays. If the target position was not reached, the PICC was adjusted until the ideal position (second to third intercostal space, within the upper one-third of the superior vena cava) was achieved.

For the experimental group treated with a modified Seldinger puncture method under ultrasound guidance, each patient’s vasculature was evaluated by ultrasound imaging with a LOGIQ Book XP-A1 ultrasonic diagnostic apparatus (GE Medical Systems Co, Ltd, China). Under ultrasound visualization, a point in the basilica vein 2–3 cm above elbow was targeted and pierced with a 21G Seldinger puncture needle (0.15-mm diameter and 0.75-mm length). The guide wire was fed into the vessel through the needle, and then the needle was withdrawn. Subsequently, the skin over the puncture point was stretched to facilitate passage of the dilator and cannula sheath assembly into the blood vessel along the guide wire, and the guide wire was pulled out. Finally, the PICC was placed into the vessel via the cannula sheath and passed slowly into the superior vena cava as shown in Additional file 3: Figure S3. The PICC position was confirmed by the bedside chest X-rays and adjusted, if necessary, exactly as described for the control group.

### Health economics evaluation

Cost-effectiveness analysis (Chen 2003) was used to calculate the cost-effectiveness ratio (CER) of each method. CER is equal to the catheterization cost (C) divided by the catheterization effectiveness index (EI), that is C/EI, with a lower CER indicating better cost-effectiveness. The total cumulative costs for patients in association with the catheterization over a 6-month period commencing from the operation was used as the cost C, including catheterization, maintenance, and complication treatment costs. The EI was a composite index (Xu 2007) that included three indicators: absence of complications, successful single-attempt puncture, and patient comfort. These three indicators were weighted (0.40, 0.25, and 0.35, respectively) in relation to the degree of importance of each as rated by four intravenous therapy experts. Thus, in our study, EI = (0.4 × non-complication rate) (0.25 × successful single-puncture rate) (0.35 × comfort rating). The incremental cost effectiveness ratio (ICER), defined as ΔC/ΔEI, was calculated as follows:

$$ {\text{ICER }} =  \left( {{\text{C}}_{ 1} - {\text{C}}_{ 2} } \right)/\left( {{\text{EI}}_{ 1} - {\text{EI}}_{ 2} } \right) $$

where the subscript 1 and 2 variables refer to the values calculated for the experimental and control methods, respectively.

### Outcome measures

Bleeding assessing, Pain scores (Ma and Ma 2011), single-attempt puncture (Shen and Li 2009), complication incidence (Zhong 2007) were recorded. The medical costs, which included catheterization cost, dressing cost, and complication treatment cost, were recorded in accordance with the standards of the *Manual of Medical Service Charge of Hunan Province* (2002 edition) (Gong 2002).

### Statistical analysis

SPSS 17.0 statistical software was used for data analysis. The secondary data entry method was used to ensure the accuracy of all data; additionally, 5% of the raw data were selected randomly for re-checking of data quality.

## Results

A total of 895 patients were recruited for the study, of which 535 were excluded and 360 were included. Half of the included patients were placed randomly into each group (N = 180 patients/group). Ultimately, 34 experimental group patients and five control group patients did not complete the study due to being discharged, transferring back to local hospitals, or declining to continue participating in the study, leaving final group sizes at 144 and 175, respectively (Additional file 1: Figure S1). The demographic and clinical characteristics of the two groups did not differ significantly (all *P* > .05) and are summarized in Table 1.

**Table 1:** General characteristics of the participants

Characteristic	Experimental group (*n* = 144)	Control group (*n* = 175)	*P*
Gender, N			.659
Males	59	76	
Females	85	99	
Age, years	57.45 ± 12.94	58.09 ± 11.98	.647
Weight, kg	53.18 ± 10.59	54.63 ± 11.72	.252
Diagnosis, N			.812
Tumor	128	157	
Non-tumor	16	18	
Payment pattern, N			.125
Medicare insurance	115	151	
Non-medicare insurance	29	24	
Insertion purpose, N			.085
Chemotherapy	123 (85%)	163 (93%)	
Others	21 (15%)	12 (7%)	

The experimental group had a higher success catheterization rate (99.3 vs. 85.1%) and a higher rate of patients describing the procedure as comfortable or very comfortable (76.6 vs. 44.7%) than the control group (both *P* < .001). The experimental group also had less bleeding (loss intra-operatively and within 24 h postoperatively), a lower average pain score, and fewer postoperative complications (Bortolussi et al. 2015; Hockley et al. 2007; Li and Chen 2015) than the control group (all *P* < .001). The costs of the procedure and overall medical costs within 6 months thereafter were higher for the experimental group than for the control group. Conversely, average posttreatment and complication treatment costs were lower for the experimental group than for the control group (Table 2).

**Table 2:** Comparison of treatment effectiveness and postoperative complications between the two groups

Parameter	Experimental group (*n* = 144)	Control group (*n* = 175)	*P*
Bleeding volume, ml			
During operation	2.41 ± 1.40	3.26 ± 1.78	<.001
24 h postoperatively	0.11 ± 0.08	0.29 ± 0.66	<.001
Pain score	2.79 ± 1.91	5.16 ± 1.43	<.001
Catheterization results			<.001
Success	143 (99.3%)	149 (85.1%)	
Failure	1 (0.7%)	26 (14.9%)	
Patient comfort level			<.001
Very comfortable	82 (57.1%)	72 (41.3%)	
Comfortable	28 (19.5%)	6 (3.4%)	
Common	18 (12.6%)	23 (13.1%)	
Uncomfortable	13 (9.2%)	20 (11.3%)	
Very uncomfortable	3 (1.6%)	54 (30.9%)	
Postoperative complications			
Soft tissue injury	1	21	<.001
Phlebitis (vein inflammation)	2	14	.005
Thrombus (CRVT/lumen occlusion)	0	4	.130
Catheter dislodgment (partial/complete)	1	23	<.001
Swelling (at exit site)	0	3	.254
Infection (local infection/CRBSI)	2	13	.015
Cost, CNY			
Insertion	2225.98 ± 0.00	1632.28 ± 0.00	<.001
Posttreatment	1106.99 ± 88.43	1233.10 ± 280.92	<.001
Complication treatment	2.99 ± 21.63	101.40 ± 273.95	<.001
6 months postoperation	3332.97 ± 88.43	2855.38 ± 280.92	<.001

As shown in Table 3, all three factors used to calculate the EI—namely comfort rate, single-puncture success rate, and non-complication rate—were significantly higher for the experimental group than for the control group. The EI (calculated from these three variables) for the experiment group was 50% higher than that EI obtained for the control group (Table 3).

**Table 3:** Comparison of EI and EI component terms between the two groups

Items	Experimental group (*n* = 144)	Control group (*n* = 175)	*P*
Comfort rate, n (%)	110 (76.6%)	78 (44.7%)	<.001
Weighted values, %	26.81	15.65	
Single-puncture success rate, n (%)	136 (94.4%)	132 (75.4%)	<.001
Weighted values, %	23.6	18.85	
Non-complication rate, n (%)	139 (97.2%)	92 (61.7%)	<.001
Weighted values, %	38.88	24.68	
EI, %	89.29	59.18	

*EI* effective index, calculated as sum of comfort rate, single-puncture success rate, and non-complications rate weighted values

As reported in Table 4, our cost-effectiveness analysis showed that the C/E values for the operation only and at 6 months postoperation were lower for the experimental group than for the control group. Furthermore, the ICER for the operation only and at 6 months postoperation also favored the experimental group (Table 4).

**Table 4:** Cost-effectiveness analysis by group

Items	Experimental group (*n* = 144)	Control group (*n* = 175)	ICER (ΔC/ΔEI)
Cost for insertion operation, CNY	2225.98	1632.28	1979.00
CER at 6 months postoperation	3732.75	4824.9	
Cost at 6 months postoperation, CNY	3332.97	2855.38	1591.97
CER for operation only	2492.98	2758.16	

*EI* effective index, values in Table 3, *C* cost, *CER* cost-effectiveness ratio = C/EI, *ICER* incremental cost effectiveness ratio

## Discussion

### Improved first-puncture success rate with US-Seldinger technique

In the experimental group, the operator used intravascular ultrasound technology to obtain a clear image of blood flow, blood vessel diameter and wall thickness, and vascular intimal smoothness (Dones et al. 2011). In this way, the blood vessels with thick, straight, abundant blood flow and few venous valves, such as the basilic or brachial vein, could be chosen directly for puncture, avoiding the cephalic veins which have more bifurcations, abnormal intimae, narrow areas, and curved areas. Thus, as would be expected, ultrasound guidance allows more precise targeting for needle puncture.

### Reduced bleeding and pain with the US-Seldinger technique

Owing to the tiny size of the needles employed, the Seldinger technique resulted in less puncture-induced local tissue damage than the traditional non-US technique. Several features of the US Seldinger technique may account for the observed reduction of blood loss including the application of pressure with gauze, skin stretching to enable fine control of blade penetration depth (within ~1 mm) and smooth passage of the guide wire, rotary insertion of the catheter sheath with the operator’s left thumb against the wing of catheter sheath, touching the frontier port of catheter sheath intra-vascularly, with the remaining four fingers applying pressure to the tip of the catheter sheath during its insertion (until 5 cm has been delivered), and immediate delivery of the catheter upon insertion of the sheath.

The mean pain score for the experimental group being 2.5 points lower than that for the control group is likely due to the use of 21G ultra-fine needles in the experimental group, versus 14G cannula needles in the control group. A larger diameter thickness would be more apt to produce pain. Furthermore, the Seldinger puncture procedure includes application of the local anesthetic procaine, whereas no anesthesia is used with the traditional method.

### US-Seldinger technique use associated with fewer complications

As shown in Table 2, 97.2% of the patients in the experimental group had no complications whatsoever, whereas only 61.7% of the patients in the control group had no complications. These results were similar to the results of other studies that showed reduced early complications, including puncture site bleeding, finger swelling, and mechanical phlebitis, with PICC placement via the US-Seldinger technique (Song et al. 2006a).

Given that prior studies have reported higher incidences of complications with cephalic vein insertion than with basilic or median cubital vein insertion (Lin et al. 2008; Song et al. 2006b), it is likely that enhanced vascular selection was an important factor in this difference.

### US-Seldinger technique associated with less discomfort

The better self-reported comfort level in the experimental group may have been related to the PICC being inserted above the elbow joint, which eliminates the sense of strain and unease during bending and stretching of the elbow joint. Additionally, as discussed above, the lower mean pain score of experimental group, attributed to use of a finer-gauge needle and local anesthesia, would be expected to contribute to the patients’ general sense of comfort.

Some patients in the control group were concerned about the catheter becoming damaged while exercising the elbow joint or loosening during nocturnal turning. Female patients in general were not particularly concerned that the catheter affected their physical appearance and were satisfied with it, which is consistent with prior studies (Shen et al. 2008; Zhang and Chen 2011).

### Comparison of cost-effectiveness between the two methods

The greater cost of the ultrasound-guided Seldinger operation relative to the traditional operation (difference of CNY 593.70) was due mainly to the costs for use of Seldinger puncture items and ultrasonography. The postoperative care costs would have been fairly close between the two groups but for the fact that patients in the control group faced greater costs in relation to treatment of complications (CNY 101.40 in the control group vs. CNY 2.99 in the experimental group). Furthermore, because catheters cannot be re-used, failed insertions in the control group (totaling 26) wasted substantial health resources (i.e. CNY 573.32, not including costs related to other materials and human resources). Catheter-related bloodstream infection and venous thrombosis complications cost more to treat in the control group (CNY 638-1654), and this difference of more than CNY (Royer 2001; Rice-Townsend et al. 2014) 1000 exceeded the experimental group’s extra insertion cost of CNY 593.70. Catheter-associated deep vein thrombosis, blood stream infection, and catheter occlusion are the main complications that increase cost and the main triggers of unplanned discontinuation of catheter use. Generally, we observed that our patients were more concerned about pain, catheterization safety, treatment success, and PICC retention time than they were about the cost of the insertion procedure, which receives great attention from medical staff.

The markedly better EI of the experimental group (89.29) relative to the control group (59.18) in our study points to a prominent difference in overall effectiveness. Moreover, our finding of a better CER for the experimental group than for the control group (CNY 3732.75 vs. 4824.9) indicates that, overall, US-Seldinger catheterization was more cost-effective than the tradition method. However, there was much factor uncertainty impacting the cost-effectiveness analysis, and it is not known how variations in these factors in practice would influence cost-effectiveness in other contexts. Notably, consistent with prior work, when only the operation costs were considered, without any influence of complication treatments, we still observed a more favorable CER for the experimental group than for the control group (CNY 2492.98 vs. 2758.16) (Saltelli et al. 2008). In practice, all medical procedures carry at least some potential risk, and it may be impossible to prevent complications entirely. Furthermore, it is impractical to fluctuate cost standards arbitrarily in practice. Hence, the cost parameters in both groups were kept stable to make assessment of cost-effectiveness as objective as possible, in accordance with the clinical reality.

In summary, although the cost of a single catheterization operation is slightly higher with US-Seldinger than with the traditional non-US trans-cannula PICC insertion method, we found that the overall cost-effectiveness of the former was better owing to a reduced complication rate. Furthermore, the US-Seldinger technique was associated with less patient discomfort and bleeding. The findings of the present randomized controlled trial are in agreement and extend prior research demonstrating the superior clinical results obtained with the US-Seldinger technique of PICC placement relative to a traditional non-ultrasound, non-Seldinger superficial PICC placement approach (Blaivas et al. 2012). Hence, these findings suggest that the US-Seldinger technique is suitable for clinical application.

## Additional files

**Additional file 1: Figure S1.** Study flowchart.

**Additional file 2: Figure S2.** Traditional PICC placement procedure. (a) Short peripheral venous catheter CNP of the vein. (b) Insertion of PICC through the short peripheral venous catheter sheath. (c) Tube fixing. (d) X-ray orientation.

**Additional file 3: Figure S3.** Ultrasound-guided modified Seldinger PICC procedure. (a) Vessel imaging. (b) Vein puncture. (c) Insertion of guide wire. (d) Withdrawal of the needle and expansion of the puncture point. (e) Catheter preparation. (f) Insertion/intubation dilator/sheath component along the guide wire. (g) Placement of dilator/catheter sheath component in the vein along the guide wire. (h) Withdrawal of the guide wire and dilator. (i) Catheter insertion into pre-intubation. (j) Withdrawal of the sheath. (k) Tube fixing. (l) X-ray orientation.

## Abbreviation

PICCs: peripherally inserted central catheters
