# Supplementary figures and images for: Cost-effectiveness analysis of ultrasound-guided Seldinger peripherally inserted central catheters (PICC)
Source: Springerplus. 2016 Dec 1;5(1):2051. doi: 10.1186/s40064-016-3698-8 (PMC5130933; doi:10.1186/s40064-016-3698-8)

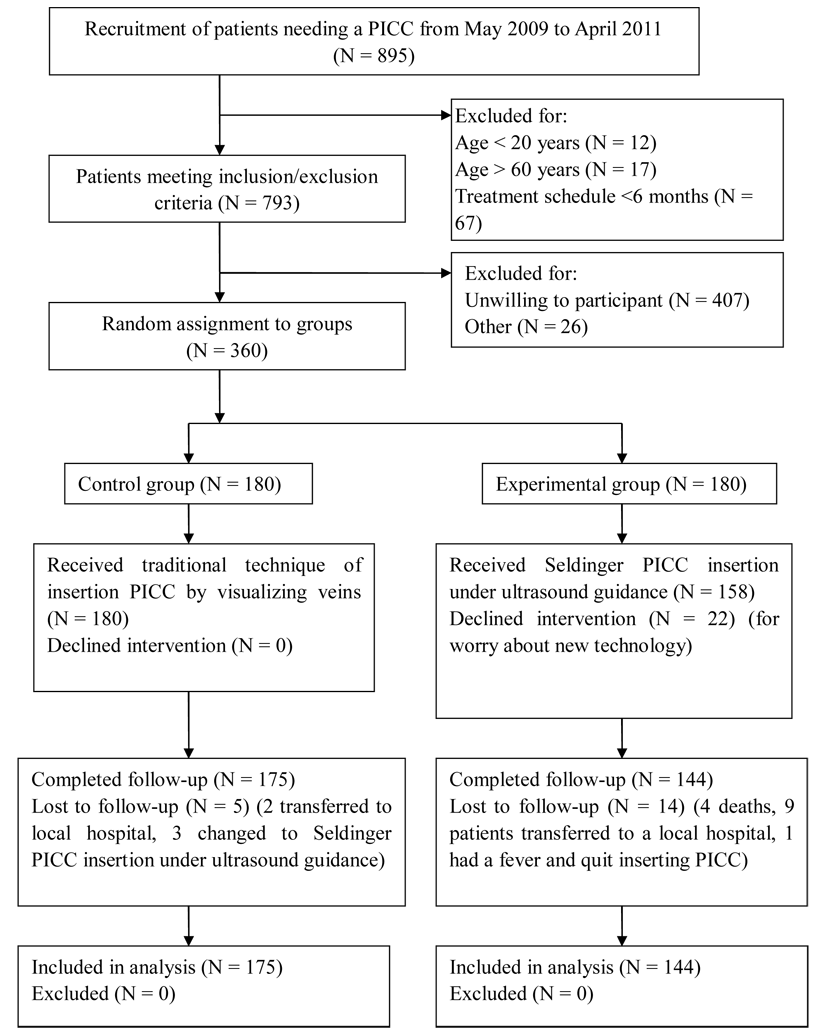

Supplement: Supplementary file 1 — Additional file 1: Figure S1. Study flowchart. [file 40064_2016_3698_MOESM1_ESM.tif]

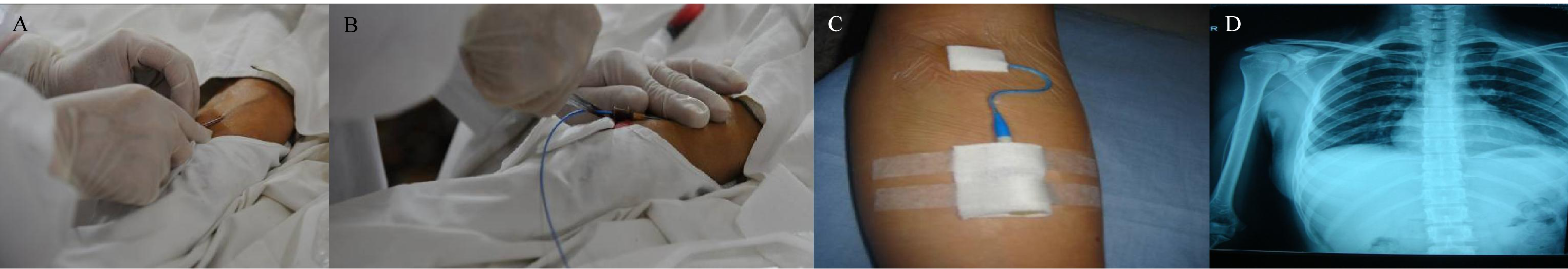

Supplement: Supplementary file 2 — Additional file 2: Figure S2. Traditional PICC placement procedure. (a) Short peripheral venous catheter CNP of the vein. (b) Insertion of PICC through the short peripheral venous catheter sheath. (c) Tube fixing. (d) X-ray orientation. [file 40064_2016_3698_MOESM2_ESM.tif]

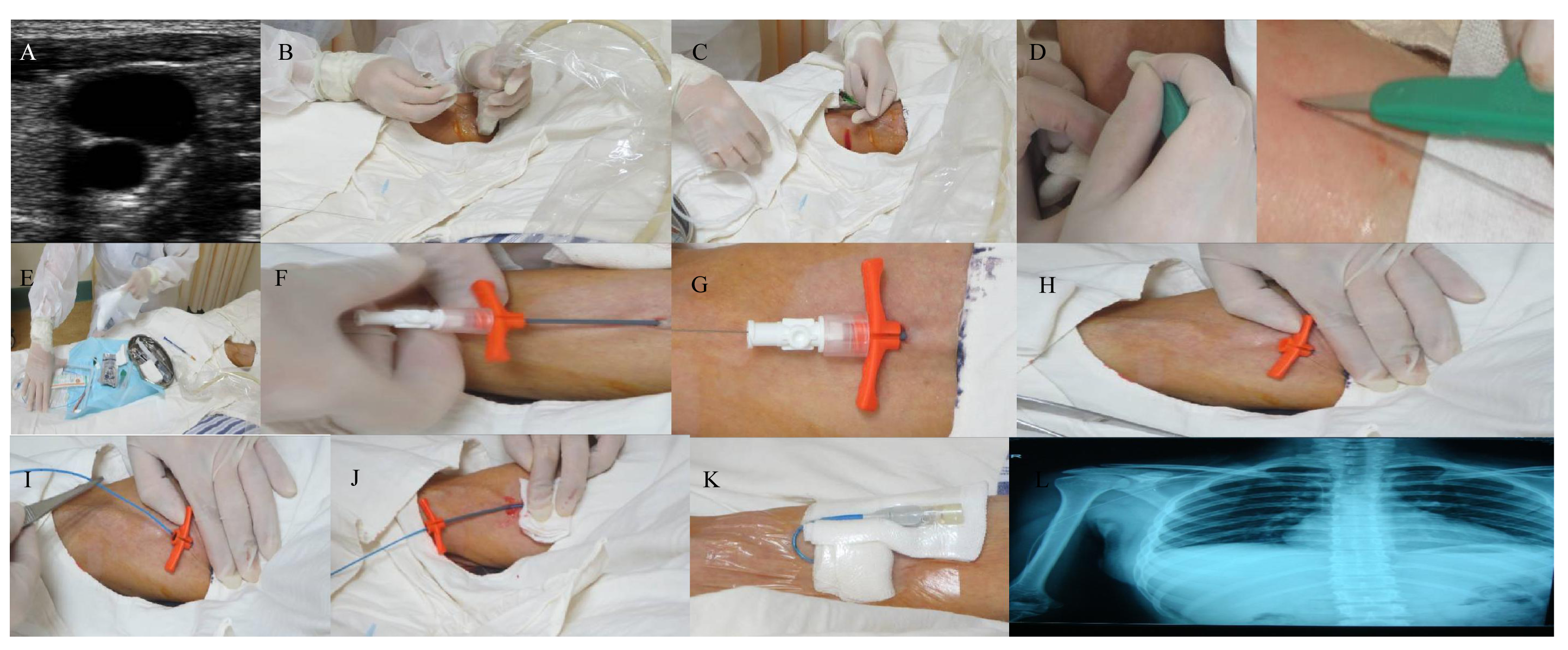

Supplement: Supplementary file 3 — Additional file 3: Figure S3. Ultrasound-guided modified Seldinger PICC procedure. (a) Vessel imaging. (b) Vein puncture. (c) Insertion of guide wire. (d) Withdrawal of the needle and expansion of the puncture point. (e) Catheter preparation. (f) Insertion/intubation dilator/sheath component along the guide wire. (g) Placement of dilator/catheter sheath component in the vein along the guide wire. (h) Withdrawal of the guide wire and dilator. (i) Catheter insertion into pre-intubation. (j) Withdrawal of the sheath. (k) Tube fixing. (l) X-ray orientation. [file 40064_2016_3698_MOESM3_ESM.tif]
